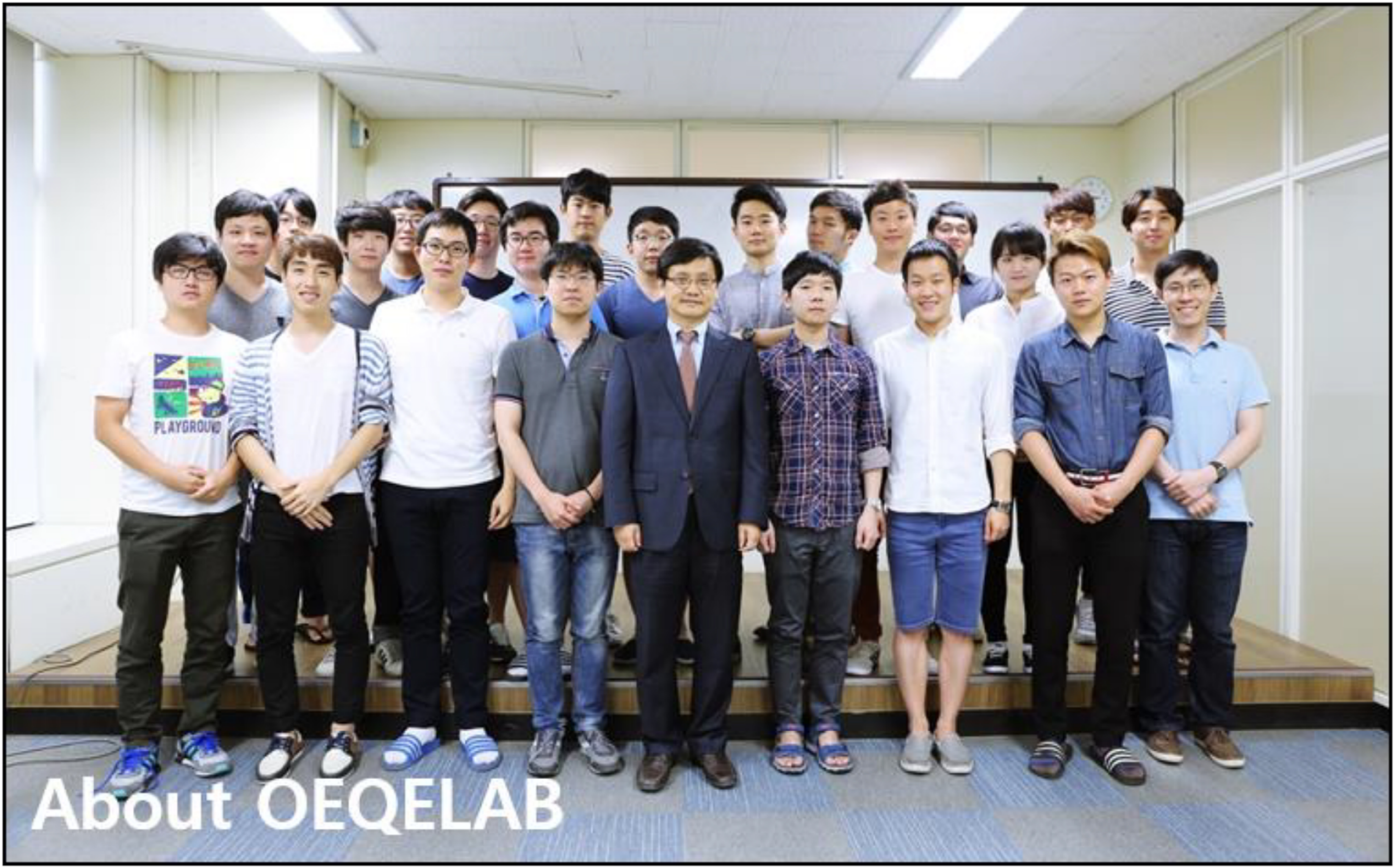# In memory of Prof. Byoungho Lee

**DOI:** 10.1515/nanoph-2023-0359

**Published:** 2023-06-23

**Authors:** Hwi Kim, Seung-Yeol Lee

**Affiliations:** Department of Electronics and Information Engineering, College of Science and Technology, Korea University, Sejong-Campus, 2511, Sejong-ro, Sejong 30019, Republic of Korea; School of Electronic and Electrical Engineering, Kyungpook National University, Daegu, Republic of Korea

**Figure:**
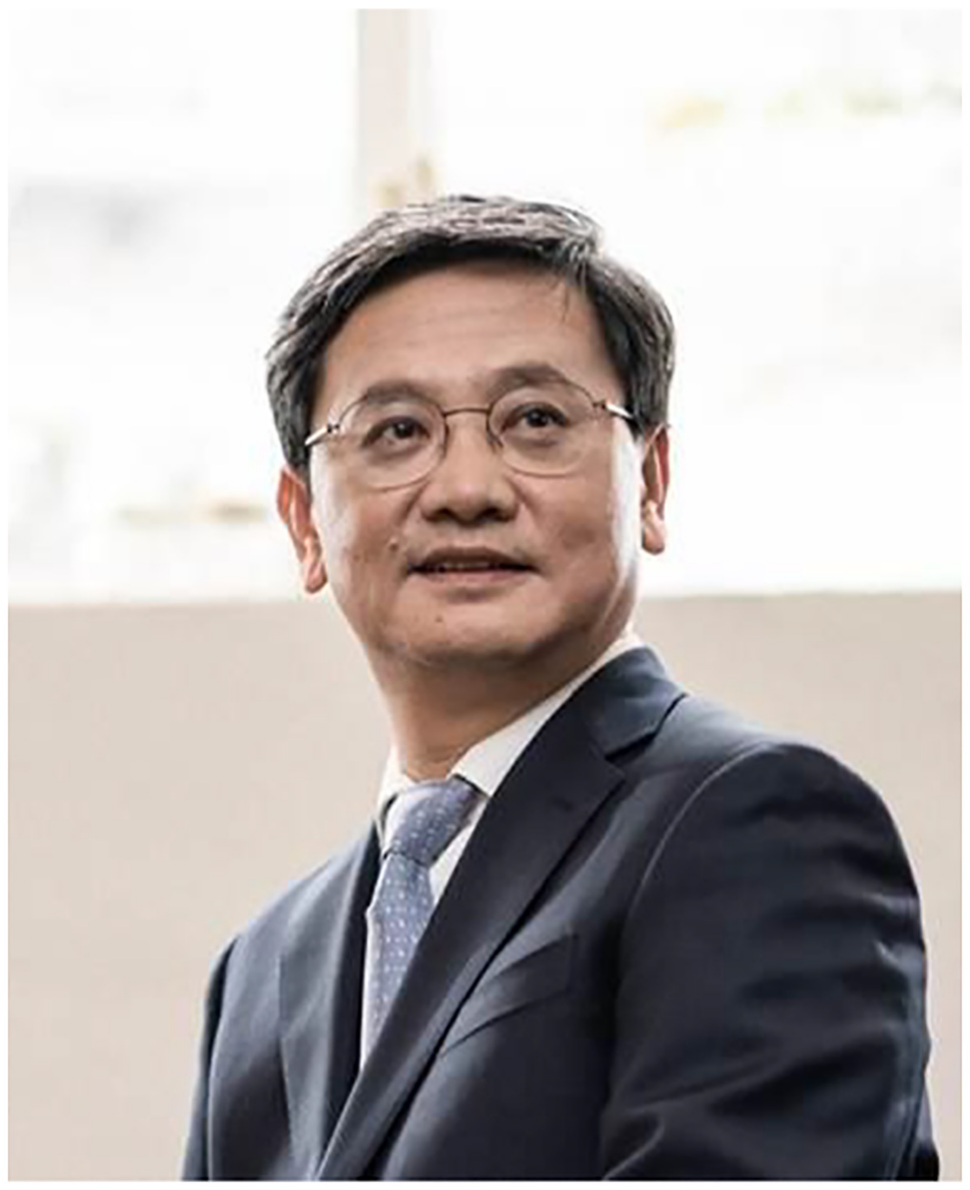


With deep reverence and admiration, we commemorate the remarkable scientific life of Prof. Byoungho Lee, an exceptional scholar who possessed confidence and humility. His inspiring passion for unlocking the full potential of nanophotonics for the future three-dimensional display technology remains unparalleled. He initiated the A3 Metamaterial forum with his outstanding colleagues, bridging leading Korean, Chinese, and Japanese researchers across Northeast Asia.

Tragically, Prof. Lee passed away on November 7, 2022, at the age of 58, leaving behind a legacy of significant contributions in optical metamaterials and metasurfaces, holographic displays, and AR/VR technology.

Born in Seoul, Korea, 1964, Lee’s lifelong thirst for knowledge was evident from an early age. His passion for science and technology led him to yearn for academic life. He earned a bachelor’s degree from the Department of Electrical Engineering at Seoul National University (SNU) and a doctoral degree from the University of California, Berkeley, USA. As a professor in the Department of Electrical Engineering at SNU, he dedicated his life to conducting fundamental research in nanophotonics and 3D display and their fusion. One of his primary research goals was to realize ultra-compact augmented displays, focusing on designing various metalenses and metasurfaces for AR/VR/hologram applications. Through his invaluable insights into metamaterials and plasmonics, he identified their technical challenges and proposed pragmatic solutions.

Lee conducted pioneering studies in the fusion of nanophotonics and augmented reality 3D display devices. As a leader of the national creative research center for active plasmonics application systems, his research led to the groundbreaking development of plasmonic metasurfaces that applied geometric phase to various metal-based surface plasmon field modulators such as vortex lenses and compact polarimeters [1], [2], [3]. Furthermore, he made a significant discovery regarding using metasurfaces to dramatically expand the viewing angles of augmented reality devices, resulting in the design of a compact near-eye display system with a wide field of view [4]. He proposed the concept of cavity apertures, which function as tiny dynamic color pixels in display and sensing applications, offering simultaneous control over the colors and intensity of visible light using the nanoaperture in the cavity and the incident light’s polarization state [5]. His research also included the active control of light within the subwavelength scale based on various phase change materials, such as VO2 and Ge2Sb2Te5, which contributed to the development of active components for next-generation micro-display panels [6, 7].

Lee’s research achievements garnered global recognition, with over 500 peer-reviewed scientific articles co-authored by him and a remarkable total citation count of 25,000 on Google Scholar. His dedication to advancing the field of nanophotonics and 3D displays inspired interdisciplinary research for ultimate meta-vision technology. As a highly esteemed scholar, he served as a committee member in the *A3 Metamaterials forum* from 2016 to 2022. His remarkable contributions were acknowledged through many fellowships from prestigious photonics and display organizations, including SPIE, Optica (formerly OSA), IEEE, and SID (the Society for Information Display). He received numerous awards, including the Presidential Young Scientist Award of Korea, Scientist of the Month Award of Korea, Jin-Bo-Jang’s National Badge of Science of Korea, and the Sudang Award. Noteworthy was his appointment as Holoknight, the Knight of Holography, in 2014 by the International Order of Holoknights [8].

Beyond his outstanding achievement as a researcher, Byoungho Lee was a mentor renowned for his humility and wit. He provided generous support to his colleagues and nurtured and inspired young scientists by offering them research and collaboration opportunities. His innovative spirit and visionary mindset were complemented by his warm and caring nature, making him a beloved teacher, leader, and friend. He guided and motivated over 100 Ph.D. and Master’s degree students, many of whom now hold prominent positions in the display and photonics communities, both in academia and industry, on the international stage.

We honor his memory and strive to carry forward his vision and passion, ensuring that his contributions continue to shape the future he dreamed. May the legacy of Prof. Byoungho Lee endure as a beacon of inspiration for the next generation.